# 901. Medical Staff and Nursing Team: Can We All Get Along by Using the Same Form?

**DOI:** 10.1093/jbcr/irag033.397

**Published:** 2026-04-06

**Authors:** Cherry Song, Abraham Houng

**Affiliations:** The Burn Center at Cooperman Barnabas, Livingston, NJ; The Burn Center at Cooperman Barnabas, Livingston, NJ

## Abstract

**Introduction:**

Rounding tools are increasingly used in critical care settings to standardize communication, enhance quality improvement, and facilitate education. At our institution, a burn ICU rounding form was adopted for daily use with critically ill patients. While primarily designed for the medical team to facilitate the residents’ presentation, nursing team was observed using parallel rounding sheets to guide patient care and handoff between shifts. This raised the question of whether transitioning nurses to the burn ICU rounding form would streamline workflow, reduce duplication and potentially decrease communication errors.

**Methods:**

A structured interview study was conducted using a convenience sample of burn ICU nurses. Questions focused on perceived utility of the burn ICU rounding form, differences compared to the nursing-specific sheet, and preferences regarding layout, content, and usability. The forms were also piloted in selected nursing handoff.

**Results:**

Participants included nurses with varying years of burn ICU experience. Nurses consistently reported that their current rounding sheet differs in focus from the burn ICU rounding form, with emphasis placed on bedside tasks and direct clinical management. The burn ICU rounding form is organized by organ system with an emphasis on capturing organ dysfunction. This information, though important, is less useful to nursing care. Much of the information overlaps, but the layout and organization of the rounding form was perceived as less practical for nursing workflows.

**Conclusions:**

Although institutional rounding form and nursing sheets contain similar information, they serve different purposes. Nurses value having their own documentation format, which supports task prioritization and individualized patient care. Streamlining to a single form may offer some efficiency gains but could compromise nursing effectiveness at the bedside.

**Applicability of Research to Practice:**

Directly applicable.

**Funding for the study:**

N/A.

